# Back to the tubule: microtubule dynamics in Parkinson’s disease

**DOI:** 10.1007/s00018-016-2351-6

**Published:** 2016-09-06

**Authors:** Laura Pellegrini, Andrea Wetzel, Simone Grannó, George Heaton, Kirsten Harvey

**Affiliations:** 1 Department of Pharmacology, UCL School of Pharmacy, University College London, 29-39 Brunswick Square, London, WC1N 1AX UK; 2 Laboratory of Neurogenetics, National Institute on Aging, National Institutes of Health, 35 Convent Drive, Bethesda, MD 20982-3707 USA; 3 Department of Neurodegenerative Disease, UCL Institute of Neurology, University College London, Queen Square, London, UK

**Keywords:** Parkinson’s disease, Cytoskeleton, Microtubule dynamics, Axonal transport, Tau, PARK genes, LRRK2, Wnt signalling, Microtubule targeting agents

## Abstract

Cytoskeletal homeostasis is essential for the development, survival and maintenance of an efficient nervous system. Microtubules are highly dynamic polymers important for neuronal growth, morphology, migration and polarity. In cooperation with several classes of binding proteins, microtubules regulate long-distance intracellular cargo trafficking along axons and dendrites. The importance of a delicate interplay between cytoskeletal components is reflected in several human neurodegenerative disorders linked to abnormal microtubule dynamics, including Parkinson’s disease (PD). Mounting evidence now suggests PD pathogenesis might be underlined by early cytoskeletal dysfunction. Advances in genetics have identified PD-associated mutations and variants in genes encoding various proteins affecting microtubule function including the microtubule-associated protein tau. In this review, we highlight the role of microtubules, their major posttranslational modifications and microtubule associated proteins in neuronal function. We then present key evidence on the contribution of microtubule dysfunction to PD. Finally, we discuss how regulation of microtubule dynamics with microtubule-targeting agents and deacetylase inhibitors represents a promising strategy for innovative therapeutic development.

## Introduction

PD is a neurodegenerative disorder characterised by significant degeneration of dopaminergic neurons within the *substantia nigra pars compacta* of the midbrain [1, 2]. It is the second most common neurodegenerative disease, with a general prevalence of 0.3 % in industrialised countries [3]. PD aetiology is complex and still poorly understood. Environmental exposure and genetic predisposition contribute to the incidence of the disease, but ageing remains the major risk factor. The prevalence increases from 1 %, in people over 60 years of age, to 4–5 % in individuals over 80 years of age [3, 4]. Most commonly, patients develop late-onset PD between the age of 60 and 70, but some cases are diagnosed before the age of 50, classified as early onset PD [5]. A number of studies suggest also higher prevalence amongst men than women, although these findings remain controversial [3]. Around 90 % of PD cases are sporadic, but the continuing discovery of several causative mutations linked to familial forms of PD may also improve understanding of the pathogenesis in sporadic cases [3, 6, 7]. Over the last 20 years, genome-wide association studies and genetic analysis identified numerous loci containing causative mutations and PD risk variants. Some of these mutations are causative for PD, others for clinically similar parkinsonian syndromes [8].

The cardinal parkinsonian symptoms of progressive bradykinesia, rigidity and resting tremor have long been described [9]. Though PD is regarded as foremost a movement disorder, there are a number of well-described non-motor symptoms, such as autonomic dysfunction, sleep disturbance, depression, cognitive decline and dementia [10]. Moreover, a variety of molecular and cellular pathways have been suggested to play a role in the pathogenesis of PD. These include, but are not limited to, accumulation of misfolded protein aggregates associated with proteasomal and autophagic dysfunction, neuroinflammation, mitochondrial damage and oxidative stress [11–14]. Development of effective interventional therapies currently poses a major challenge partially due to the diversity of affected molecular pathways and lack of consensus on which mechanisms might constitute the primary insult leading to PD. Currently, dopaminergic transmission is partially restored in a majority of newly diagnosed patients by administration of the neurotransmitter precursor levodopa [15]. Although there are temporary improvements in symptoms, the levodopa therapy tends to lose efficacy over time and often leads to debilitating side effects [15–17]. Long-term studies have shown that early co-administration of bromocriptine may delay the onset of side effects [18, 19]. Despite having produced significant improvements in PD patients, particularly at early stages, levodopa therapy remains a non-disease-modifying approach.

Mounting evidence suggests that deregulation of neuronal cytoskeleton function constitutes a key insult during the pathogenesis of neurodegenerative diseases. Understanding the fine regulation of cytoskeletal components particularly important for microtubule dynamics, axonal transport and synaptic function is a crucial first step to unravel dysfunctional mechanisms leading to neurodegeneration. Microtubules form intracellular transport highways, which allow trafficking of molecular cargo along axons to facilitate neuronal function. Dynamic reorganisation of microtubules has long been known to mediate essential aspects of cellular homeostasis, including mitosis, vesicular transport, organelle and protein trafficking as well as maintenance of structural integrity. Microtubule dysfunction is increasingly viewed as important contributor to PD pathogenesis. Lewy bodies, a classical histological feature of PD, have been found to contain tubulin and neurofilaments, key elements of the neuronal cytoskeleton [20–22]. Moreover, multiple lines of evidence suggest that the PD-associated proteins α-synuclein, parkin, PINK1 and the Leucine-rich repeat kinase 2 (LRRK2) may modify microtubule stability [23–26]. Here, we outline the role of microtubules within neuronal function, their post-translational modifications and associated proteins. We further explore the evidence linking microtubule dysfunction to PD, before discussing recent advances in the possible use of microtubule targeting therapeutics for neurodegenerative diseases.

## Microtubules

### Structural overview

**Fig. 1:**
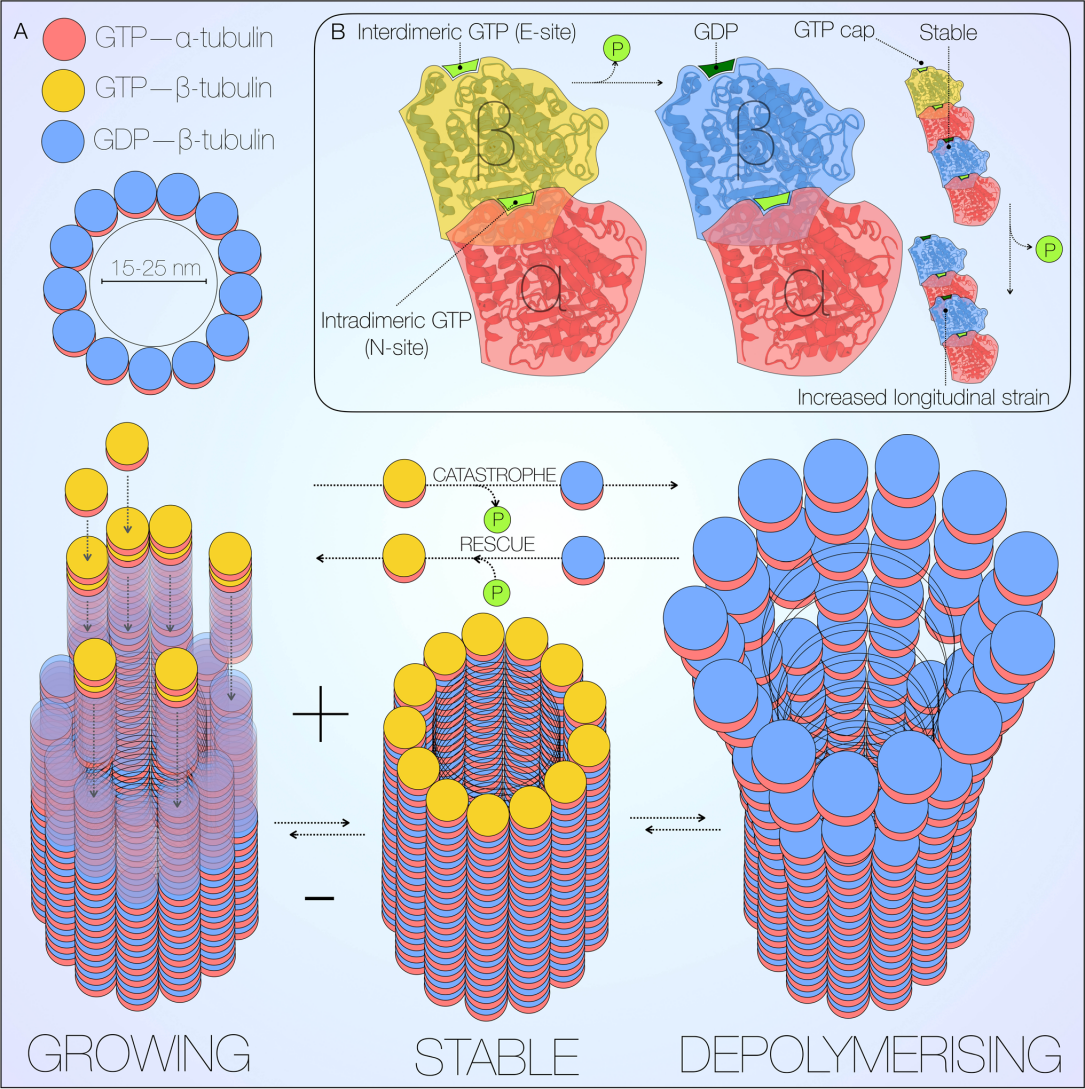
Microtubule structure and dynamic instability. Microtubules are long, dynamic, cylindrical polymers with a diameter of 15–25 nm. Each microtubule is composed by 13 protofilaments of α/β-tubulin heterodimers, which assemble forming a tubular structure (**a**). At microtubule plus ends, GTP-bound β-tubulin caps growing filaments, attracting further GTP-bound α/β-tubulin heterodimers. Upon binding of a new heterodimer, β-tubulin GTP on the filament undergoes hydrolysis at the ‘exchangeable’ site (E-site), ensuring the GTP cap remains on the first heterodimer (**b**) [27, 36]. The *cyclic* incorporation and loss of GTP β-tubulin, stochastically alternates growing (rescue) stable and depolymerising states (catastrophe). The ‘non-exchangeable’ site (N-site) is occupied by the intradimeric, α-tubulin-bound GTP, and ensures structural stability [35, 36]. *Crystal structure* of tubulin from PDB [http://www.rcsb.org/pdb/pv/pv.do?pdbid=1TUB&bionumber=1], mechanism adapted from [36]

Microtubules are highly dynamic intracellular polymers. In close association with F-actin, they form an integral component of the neuronal cytoskeleton [27]. As well as controlling neuronal morphology, the cytoskeleton also regulates trafficking and cell division [28]. Microtubules are assembled modularly from α/β-tubulin heterodimers, of which six and seven isoforms are currently described, respectively [27, 29]. This composition generates polarity in microtubules, with growth and shrinkage occurring at the plus end [27, 30], although minus end polymerisation has also been observed [28]. At the core of this process is a tight balance of growth and shrinkage referred to as dynamic instability (Fig. 1a) [31, 32].

Primary assembly of GTP-bound α/β-tubulin heterodimers generates linear protofilaments bound to a template structure known as the γ-tubulin ring complex [33, 34]. The γ-tubulin complex caps the microtubule minus end resulting in microtubule growth from the plus end. The intradimeric, α-tubulin-bound GTP occupies the ‘non-exchangeable’ site (N-site), and exerts a fixed structural role [35, 36]. Heterodimers are bound to the growing plus end via α-tubulin interaction with an interdimeric GTP molecule, bound to β-tubulin at the ‘exchangeable’ site (E-site) (Fig. 1b) [27, 36]. The GTP cap must remain at the foremost plus end for efficient assembly, and is therefore hydrolysed to GDP upon binding of a new GTP-bound heterodimer, effectively shifting upwards in position. Protofilaments then laterally assemble into a circular array with 13-fold symmetry, resulting in a tubular structure approximately 15–25 nm in diameter (Fig. 1a). Hydrolysis of the E-site GTP cap results in a longitudinal strain along the microtubule, facilitating disassembly of the protofilaments and release of free α/β-tubulin heterodimers [36, 37]. Although the exact structural nature of this process has long remained elusive, it has recently been shown that loss of the E-site GTP leads to compression of the interdimeric interface, with resultant instability [36]. The GTP-dependence inherent to tubulin polymerisation is primarily responsible for its dynamic nature. Self-nucleation of microtubules in the presence of GTP is observable in vitro [38], although multiple accessory proteins facilitate this highly energy dependent process intracellularly [28, 29, 39].

**Fig. 2:**
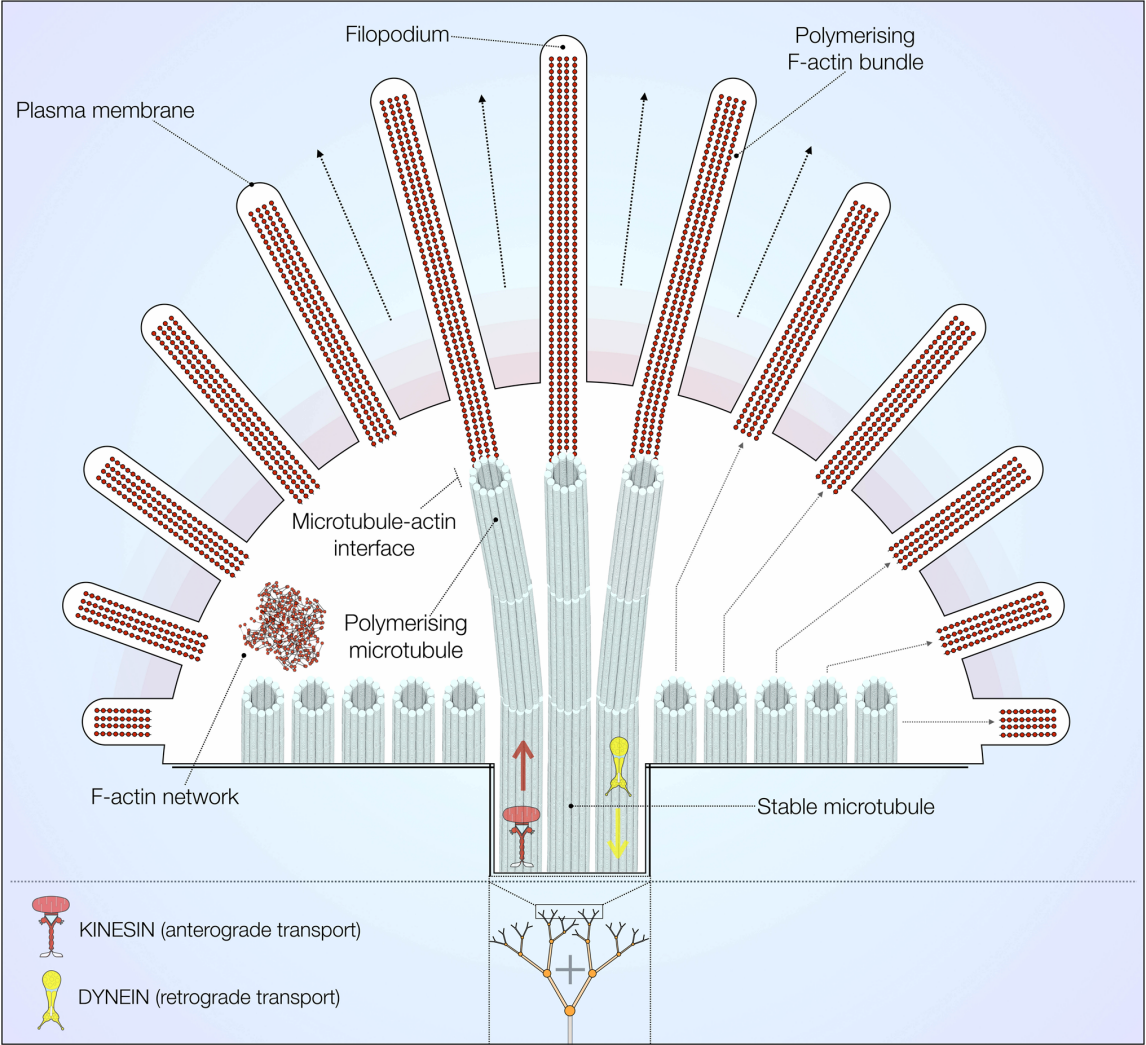
Cytoskeletal distribution in growth cones. At the growth cone, F-actin extends the plasma membrane forming filopodia and polymerises in bundles. These F-actin bundles associate with dynamic and growing microtubules to promote axon outgrowth. Stable microtubule bundles are present in the *centre* of growth cones

In neurons, microtubules, actin filaments and neurofilaments compose the cytoskeleton, maintaining cell polarity, architecture and morphology [40]. Microtubule bundles are spread throughout the soma, dendrites and axon, connecting these different compartments [30]. The axonal shaft displays uniform plus end orientation, while dendrites possess more variable combinations [27, 30]. Microtubules also have a prominent role in growth cone function, a highly dynamic process directing axonal growth during neuronal development (Fig. 2). The complex process of axon outgrowth requires spontaneous electrical activity provided by calcium and sodium channels [41–43] as well as a set of specific growth cone receptors interpreting external guidance cues [44]. Activation of growth cone receptors triggers intracellular signalling pathways leading to the formation of a ‘molecular clutch’ [45]. This complex links receptors to the F-actin cytoskeleton present in the periphery of growth cones [45, 46]. Here, dynamic microtubules are tethered to F-actin filaments through a variety of cross-linking proteins [27, 47]. Examples include the plus-tip interacting protein (+TIP) class, which mediate axonal extension both in *Drosophila* and mouse cortical neurons through the subfamily of spectraplakins [48, 49]. The centre of growth cones is composed of stable microtubule bundles, which are surrounded by a dynamic F-actin network [50]. The cytoskeletal composition within growth cones determines their shape and movement during development. F-actin filaments exert pressure on the plasma membrane via a ‘treadmilling’ mechanism of directional assembly and disassembly [27, 44]. At the same time, microtubules are guided and effectively pulled along the direction of F-actin polymerisation, both outwardly and in retrograde flow [44]. The combination of these processes results in remodelling and/or extension of the neuronal plasma membrane [51, 52]. The importance of axonal outgrowth and growth function for late-onset neurodegenerative diseases is currently unclear but it should be noted that changes in neurite outgrowth and growth cone morphology in genetic PD models have been observed, for example, in *LRRK2* PD models [23, 53–55].

### Post-translational modifications of microtubules

**Fig. 3:**
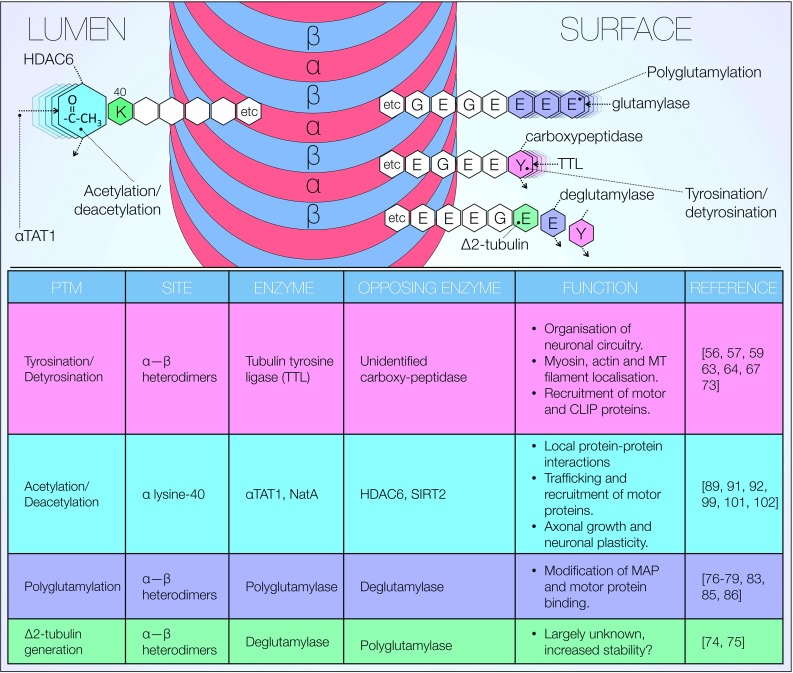
Post-translational modifications of tubulin. Overview of post-translational modifications (PTMs) of tubulin including target sites, associated enzymes and known effects. Microtubules in subcellular compartments are functionally and structurally distinguished, with stable and dynamic axonal microtubules presenting differential PTM distributions

Microtubule dynamics and therefore stability are primarily regulated through a number of post-translational modifications (PTMs) of tubulin, both in its heterodimeric and protofilament conformation (Fig. 3). Multiple modifications often occur simultaneously and vary depending on cell type and subcellular localisation. Such complexity results in the generation of microtubules with spatially and temporally dependent characteristics. PTMs also regulate microtubule binding to a high number of proteins, further contributing to tight regulation of neuronal cytoskeletal dynamics. Here, we focus on current evidence surrounding some of the well-described PTMs which have been observed to play a role in microtubule function: tyrosination, polyglutamylation, acetylation as well as generation of Δ2-tubulin (Fig. 3) [29]. We would like to stress that the significance of the findings discussed below for physiological and pathogenic events remains controversial in nature.

In 1975, Arce et al. identified tyrosination as the first PTM of tubulin [56], characterising the incorporation of tyrosine onto the α-tubulin C-terminal in rat brain homogenates [56]. The reversibility of this reaction was described by Hallak et al. shortly after [57]. Interestingly, α-tubulin is tyrosinated in its nascent state [58], with the cyclic addition and removal of C-terminal tyrosine (detyrosination) constituting its primary modification. The carboxypeptidase responsible for α-tubulin detyrosination remains unidentified at present. Moreover, its tyrosinating counterpart tubulin tyrosine ligase (TTL) is far better characterised [59–62]. TTL acts exclusively on α/β heterodimers and is thought to tyrosinate all soluble tubulin prior to microtubule polymerisation. Thus, all assembling microtubules are solely composed of tyrosinated tubulin. This notion is strongly suggestive of correct microtubule assembly being dependent on tyrosination. In accordance, selective knockout of TTL expression has been shown to induce death in mice at 1 day of age [63]. TTL ablation resulted in widespread disturbance of neuronal organisation, with prominent disruption of cortico-thalamic circuitry acting as a likely candidate for lethality [63]. TTL-null cultured neurons also exhibit morphological abnormalities, including excessive neurite outgrowth with premature axon differentiation [63]. Similarly, Marcos et al. reported that TTL knockout in precerebellar neurons results in aberrant filopodia projections and enlarged growth cones, with significantly altered localisation of myosin and actin filaments as well as microtubules [64]. Unsurprisingly, these cellular areas are rich in tubulin polymerisation.

At the molecular level, tubulin lacking a C-terminal tyrosine functional group exhibits a greater capacity for recruitment of motor proteins over its tyrosinated counterparts. Kinesins, a class of molecular motors responsible for trafficking along microtubules, have been reported to bind the exposed glutamate subunit of detyrosinated microtubules with 2.8-fold higher binding affinity relative to tyrosinated microtubules [65]. Immunofluorescence imaging of modified kinesin heavy chain (KIF5C) has shown that kinesin-1 preferentially binds and traffics along detyrosinated microtubules in neuronal cell lines [66]. In 2009, Konishi and Setou proposed that the relative abundance of detyrosinated in comparison to tyrosinated tubulin in neurons may act as a candidate mechanism for directing kinesin-1 to specific neuronal cellular compartments. Kinesin-1 is typically confined to axonal regions of neurons. Konishi et al. reported that inhibition of TTL via siRNA knockdown in rat hippocampal neurons resulted in a decrease in tubulin tyrosination, and a subsequently broader distribution of microtubule-associated kinesin-1 to both axonal and dendritic cellular compartments [67].

Microtubules with long turnover times appear enriched with detyrosinated tubulin, associating detyrosination with increased microtubule stability [68]. Intriguingly, whilst detyrosination might be necessary for increased stability, it does not appear to be a sufficient condition [69, 70]. +TIPs have also been shown to differentially bind to detyrosinated microtubules. In mammalian fibroblasts, cytoplasmic linker protein (CLIP) 170 shows higher affinity for tyrosinated microtubules, whereas detyrosination of α-tubulin inhibits CLIP170 binding [71, 72]. These findings are strongly suggestive of tyrosination as a key mechanism mediating microtubule protein–protein interactions. More recent evidence suggests that detyrosinated microtubules may play a modulatory role in striated muscle mechanotransduction [73]. Moreover, detyrosinated tubulin may upregulate X-ROS signalling, a transduction pathway through which reactive oxygen species (ROS) are produced in response to mechanical stress. In Duchenne muscular dystrophy (DMD), a rare X-linked condition, alterations to microtubule dynamics lead to detrimental enhancement of X-ROS signalling with downstream disruption of calcium signalling. Kerr et al. demonstrate that in vivo inhibition of detyrosination through TTL overexpression reduces stress induced X-ROS and calcium signalling in a DMD mouse model [73].

Detyrosination of α-tubulin exposes the C-terminal glutamate which is further removed by the enzyme deglutamylase. Deglutamylation subsequently generates the Δ2-tubulin isoform. Removal of the TTL binding, C-terminal glutamate residue renders this tubulin isoform resistant to tyrosination. In contrast to detyrosination, this PTM is irreversible. Despite Δ2-tubulin representing ~35 % of total brain tubulin, its functional role remains largely unclear [74]. An early characterisation study localised Δ2-tubulin to early differentiating neurons and growth cones [75]. Δ2-tubulin is also largely confined to highly stable structures such as centrosomes and primary cilia. Similar to tyrosination, however, removal of C-terminal glutamate might simply correlate with enhanced microtubule stability [75].

First described in 1990, polyglutamination is a major PTM capable of generating further diversity in tubulin [76]. Employing a combination of HPLC and mass spectrometry on mammalian brain, Eddé et al. observed the addition of glutamate residues close to the C-terminal end of α-tubulin [76]. Shortly thereafter, several groups demonstrated independently that this modification also occurs on β-tubulin [77–79]. Glutamination of both α and β tubulin occurs over two phases. First, ‘initiation’ reaction catalyses the formation of a covalent bond conjoining the γ-carboxyl group of the modified glutamate and the amine group of the added glutamate residue. Glutamate residues are subsequently incorporated as part of a secondary ‘elongation’ reaction to form acidic side chains of varying length [80]. Polyglutamylases, driving polyglutamination, are large proteins containing TTL-like (TTLL1) catalytic subunits [81]. Polyglutamylase subunit 1 (PGs1) has been identified through immunoprecipitation assays with mouse brain tubulin [82]. Although PGs1 is not directly linked to enzymatic activity, it is significantly enriched in hotspots of polyglutaminated microtubules—namely mitotic spindle, neuronal microtubule, axonomes and centrioles [80, 83, 84]. As such, PGs1 has been suggested as adaptor for recruitment of a larger polyglutamylase complex. The ROSA22 mouse, which exhibits loss of functional PGs1 activity, may offer indirect insight into the role of polyglutamylated microtubule [83, 84]. These mice display significantly reduced microtubule polyglutamylation in neurons alongside abnormal sperm flagella, heightened intermale aggressive behaviour and a reduction in body fat content [84]. Subsequent in-depth analysis of ROSA22 mutants revealed decreased binding of α-tubulin with several MAP and kinesin-3 motor proteins, although kinesin-1 and kinesin-2 binding appeared unaltered [83]. Similar to other tubulin PTMs, it is likely that polyglutamination impacts trafficking and microtubule dynamics through differential recruitment of molecular motor proteins and MAPs [85, 86].

Acetylation has long been considered to be specific to α-tubulin, occurring on the luminal surface of microtubule protofilaments at the lysine 40 residue [87]. More recently, however, Chu et al. identified the lysine 252 residue of β-tubulin as a novel acetylation site. This PTM is catalysed by the acetyltransferase San at the polymerisation interface [88]. At present, however, evidence remains limited as to the functional significance of β-tubulin acetylation. Interestingly, cryo-electron microscopy analysis of protofilament distribution indicates that α-tubulin acetylation does not appear to alter microtubule architecture or α-tubulin confirmation significantly. Howes et al. suggest instead that acetylation might regulate luminal protein–protein interactions within microtubules [89]. Deacetylase and acetyltransferase enzymes are considered to be responsible for tight regulation of α-tubulin de-/acetylation, respectively. In 2002, Hubbert et al. first identified histone deacetylase class II (HDAC6) as an α-tubulin deacetylase [90]. The authors observed in vivo α-tubulin deacetylation following HDAC6 overexpression [90]. Conversely, siRNA-mediated HDAC6 knockdown enhances lysine 40 acetylation [90]. These findings were further corroborated by Zhang et al., who reported a direct interaction between HDAC6 and β-tubulin in a yeast-two hybrid assay. HDAC6 mediated α-tubulin deacetylation was also observed in mouse embryonic stem cells within the same study [91]. It is important to point out that histone deacetylases are not tubulin-specific enzymes. They additionally target chromatin with modulatory effects on chromosomal function. This notion should be considered when assessing off-target effects of pharmacological agents affecting deacetylation. Sirtuin 2 (SIRT2), an additional tubulin deacetylase has been shown to interact with soluble heterodimers as well as assembled microtubules [92]. Interestingly, the oxidative neurotoxin 6-hydroxydopamine (6OHDA) was shown to influence SIRT2 activity and microtubule dynamics through more than one mechanism, leading to impaired nuclear import of certain transcription factors [93, 94]. Consistently, altered subcellular localisation of transcription factors has been reported in post-mortem PD brains [93, 95, 96].

Acetyltransferase activity, responsible for α-tubulin acetylation, was first described in *Chlamydomonas flagellar* [97]. In mammalian cells, acetylation is reportedly catalysed by α-tubulin acetyltransferase 1 (αTAT1) or by n-α-acetyltransferase A (NatA) [98]. Despite the available evidence, the exact mechanism by which acetyltransferases and deacetylases gain access to lysine 40 is not fully understood. Nogales et al. suggested slow diffusion of acetyltransferase down the lumen of assembled microtubules as a candidate mechanism [87]. An alternative model proposes that variability in lateral protofilament interactions may result in transient, localised openings of the microtubule wall. This model has been likened to a ‘breathing’ mechanism following cryo-electron microscopy observation of small structural defects in microtubule walls [99, 100].

Akin to tyrosination, acetylation has been implicated in control of cellular trafficking, with particular relevance to recruitment and activity of motor proteins [90, 101]. Reed et al. reported that loss of α-tubulin acetylation modifies kinesin-1 binding and trafficking activity in vitro [102]. Increased α-tubulin acetylation is also observed during axogenesis, suggesting its potential role in axonal growth and neuronal plasticity [101].

### Microtubule-mediated axonal transport

Microtubules form intracellular highways along which organelles, vesicles and proteins are constantly trafficked. The unique architecture of neurons, set apart by their long axons and dendrites, has posed the evolutionary need for a tightly organised microtubule network able to ensure efficient long-range transport. Unsurprisingly, altered microtubule regulation was shown to be an early event in neurodegenerative diseases associated with axonal transport defects [103–108].

**Fig. 4:**
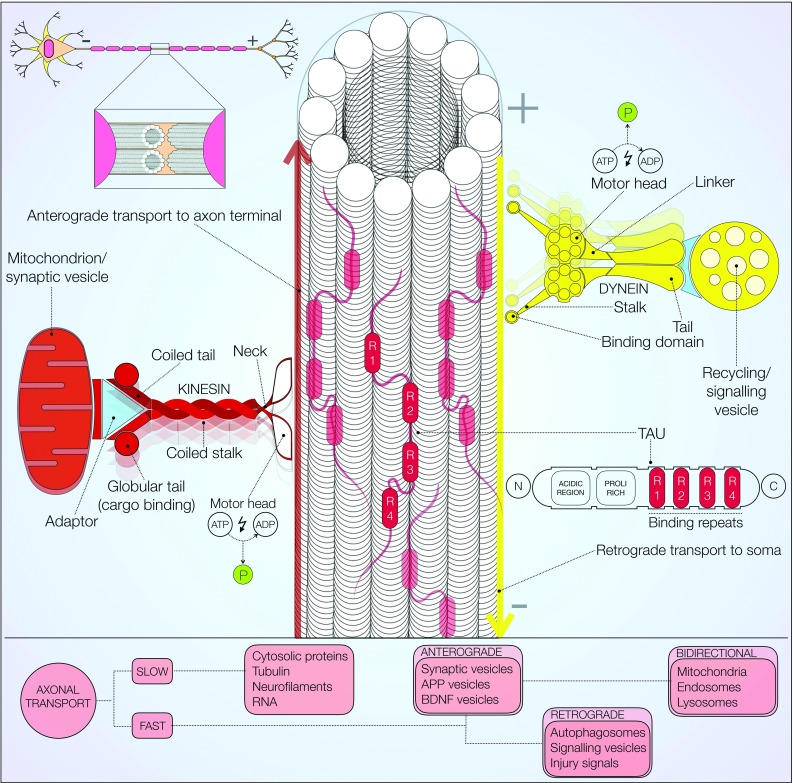
Microtubule-mediated axonal transport. Axonal microtubules are specialised in transmitting vesicles and other cargo via molecular motors. Microtubule organisation and modulation by MAPs (such as tau) also aids transport. Kinesin (*left red*) performs anterograde transport to axonal terminals. Dynein (*right yellow*) moves retrogradely towards the cell body. Fast and slow axonal transports drive organelles, vesicles and proteins along the axon. A tight balance of anterograde, bidirectional and retrograde transport is required to avoid either accumulation or depletion of cellular components [40]

Axonal transport is chiefly described as the active movement of subcellular structures along neuronal microtubules (Fig. 4) [109, 110]. In a 1948 seminal study, Weiss and Hiscoe first demonstrated its existence in chicken sciatic nerves [111]. Correct sorting of cargos and organelles requires tight regulation of this cellular mechanism at multiple levels [112]. Axonal transport is classified into fast and slow, according to cargo speed. Furthermore, directionality of movement away from, or towards the soma is used to distinguish anterograde from retrograde axonal transport, respectively [113]. ATP dependent motor proteins and a multitude of kinases regulate this complex process. Kinesins, first purified in 1985 from the squid axoplasm, are a large family of motor proteins essentially involved in anterograde transport [114]. Structurally, kinesins are cytosolic proteins with two globular heads, binding ATP and microtubules, connected to a cargo binding tail domain (Fig. 4, left). The remarkable binding specificity of kinesins arises from an extremely diverse population of tail domain binding motifs. The ‘stepwise’ movement of kinesins along microtubules is an energy dependent process requiring ATP hydrolysis [115]. Dynamic association and dissociation of these molecular motors is also dependent on local microtubule concentration, the presence of kinases and adaptor proteins [113, 116].

Anterograde transport is associated with axonal growth and synaptic vesicle replenishment [117]. Synaptic vesicles, dense core vesicles, brain-derived neurotrophic factor (BDNF) containing vesicles and amyloid precursor protein (APP) exemplify the heterogeneity of cargo actively trafficked along the axon via anterograde transport [40]. The kinesin-3 family, particularly the motor proteins KIF1A and KIF1B, is known to be involved in this transport modality [118]. Fast anterograde transport is responsible for movement of vesicles emerging from the Golgi, at an average speed of 200–400 mm per day [40]. Administration of transport blocking antimitotic agents, namely colchicine and vinblastine, has been employed to demonstrate the essential role of microtubules in axonal transport [119]. By contrast, cytosolic proteins and cytoskeletal polymers such as neurofilaments and tubulin itself are shuttled at much slower speeds of 0.1–3 mm per day [113].

Retrograde transport, primarily mediated by cytoplasmic dyneins (Fig. 4, right), is associated with intracellular recycling and injury signalling [120]. Dynein is composed of two heavy chains, intermediate, light intermediate and light chains [40]. Large microtubule binding protein complexes (MBPs) such as dynactin are responsible for tethering cargo to dyneins. Additionally, an array of adaptor proteins, of which lissencephaly-1 and huntingtin are prime examples, contributes to this process [113]. A number of +TIPs and end binding proteins, such as EB1 and EB2, are recruited to the highly dynamic microtubule plus ends thus facilitating active loading of dynein-cargo complexes [121]. Signalling endosomes, autophagosomes and injury signalling molecules are transported retrogradely towards the cell soma [120]. Microtubule instability and impaired axonal transport have been suggested to cause incomplete fusion of autophagosomes with lysosomes leading to autophagic vesicle accumulation [122]. Arduino and others also reported defects in autophagic vesicle mobilisation towards lysosomes due to disruption of microtubule dependent trafficking [123]. Overall, axonal transport regulates the functional distribution of several proteins through remarkably diverse cargo binding specificity. This notion also implies that a large variety of disease-associated proteins are likely to interact with microtubules.

### The role of tau in health and disease

Mutations and variants in the *MAPT* gene, encoding the microtubule-associated protein tau, as well as deposition of misfolded tau as neuropathological correlate have been linked to several neurodegenerative diseases, such as familial frontotemporal dementia with parkinsonism linked to chromosome 17 [124], progressive supranuclear palsy [125], chronic traumatic encephalopathy [126], amyotrophic lateral sclerosis (ALS) [127], Alzheimer’s disease (AD) and PD [121–123, 128, 129].

Microtubule dynamics are heavily dependent on an array of microtubule-associated proteins (MAPs) selectively and transiently decorating axonal microtubules. Both microtubule orientation and MAP subset are determinants of transport directionality and organelle sorting. Accordingly, axons and dendrites differ by their MAP subset. An important example is the differential distribution of two well-described MAPs: MAP2 and tau. While MAP2 is predominant in dendritic microtubules, tau is relatively more abundant in axons [30].

Tau is encoded by the *MAPT* gene located on chromosome 17q21 [130]. It associates with axonal microtubules to promote stability, although unbound tau is also found in the cytosol [131, 132]. In the human brain, tau isoforms ranging from 352 to 441 amino acids are generated through alternative splicing [131].

Tau protein structure can be divided into a highly acidic N-terminal projection domain, a central proline-rich region and a C-terminal domain. The N-terminus interacts with other cytoskeletal elements and the plasma membrane. The C-terminus usually contains three to four highly homologous 31-32 amino acid repeats functioning as binding domains [133]. Amongst these, the KXGS motif is critical for microtubule interaction as well as regulation of tau folding and aggregation. Furthermore, the tau N-terminal projection domain has long been proposed to affect axonal microtubule spacing and diameter [134]. Recent high-resolution in vitro polymerisation studies, suggest that tau may also influence microtubule-actin network cross-linking [135]. It was further shown that tau promotes tubulin polymerisation and actin bundling in vitro [134, 136]. Microtubule assembly is heavily dependent on the tau phosphorylation state; given a dephosphorylated tau conformation is necessary for polymerisation [137]. Consequently, research efforts have focused on the identification of tau regulatory kinases and phosphatases influencing microtubule binding. Most described tau kinases are proline directed protein kinases including glycogen synthase kinase 3β (GSK3β), MAP kinase (MAPK), cdc2 and cdk5. Specifically, serine 262 and threonine 231 phosphorylation at the microtubule binding site significantly reduces microtubule binding resulting in filament disassembly [136, 138].

Perhaps expectedly, tau actively participates in axonal transport and neurite outgrowth [139, 140]. Through competition for microtubule binding sites with molecular motors, it may in fact prevent or reduce organelle and cargo trafficking [141]. PTMs of tau, reportedly phosphorylation and acetylation [142], exert a modulatory action on tau function, thus influencing microtubule stability. Mass spectrometry analysis identified a lysine-rich region in the microtubule binding site as a key acetylation site [142]. In the same study, both acetylation and phosphorylation appear to induce tau release from microtubules, in turn leading to an enriched pool of cytosolic tau prone to aggregation. In addition, loss of tau acetylation, an event enhancing tau phosphorylation, has been observed in AD patients and a murine model of tauopathy [143]. Studies in transgenic models expressing mutant tau suggest that loss of tightly regulated phosphorylation state results in microtubule destabilisation and abnormal tau aggregation [144, 145]. Intracellular tau accumulation can generate inclusions termed paired helical filaments (PHF) further assembled in neurofibrillary tangles (NFT) in AD [146, 147]. In addition to AD, hyperphosphorylated tau aggregates are a classical feature in a number of neurodegenerative diseases [128, 132, 133, 148]. In the context of PD, Lewy bodies are well-known to contain tau and α-synuclein, a notion suggestive of a pathological interaction between the two proteins [149]. Accordingly, a direct tau-α-synuclein interaction has previously been reported [150]. In addition, protein kinase A and GSK3β have been proposed as kinases mediating α-synuclein-dependent tau phosphorylation [151, 152]. This process may result in microtubule depolymerisation and reduced axonal transport.

Mutations in *MAPT* have been described in frontotemporal dementia (FTD) with Parkinsonism and progressive supranuclear palsy [153, 154]. The importance of tau in neurodegeneration was further supported by genome-wide association studies identifying *MAPT* as risk factor for sporadic PD [154, 155]. In addition, the H1 *MAPT* haplotype is significantly associated with increased susceptibility to PD [153, 155].

## Microtubule dysfunction in Parkinson’s disease

**Fig. 5:**
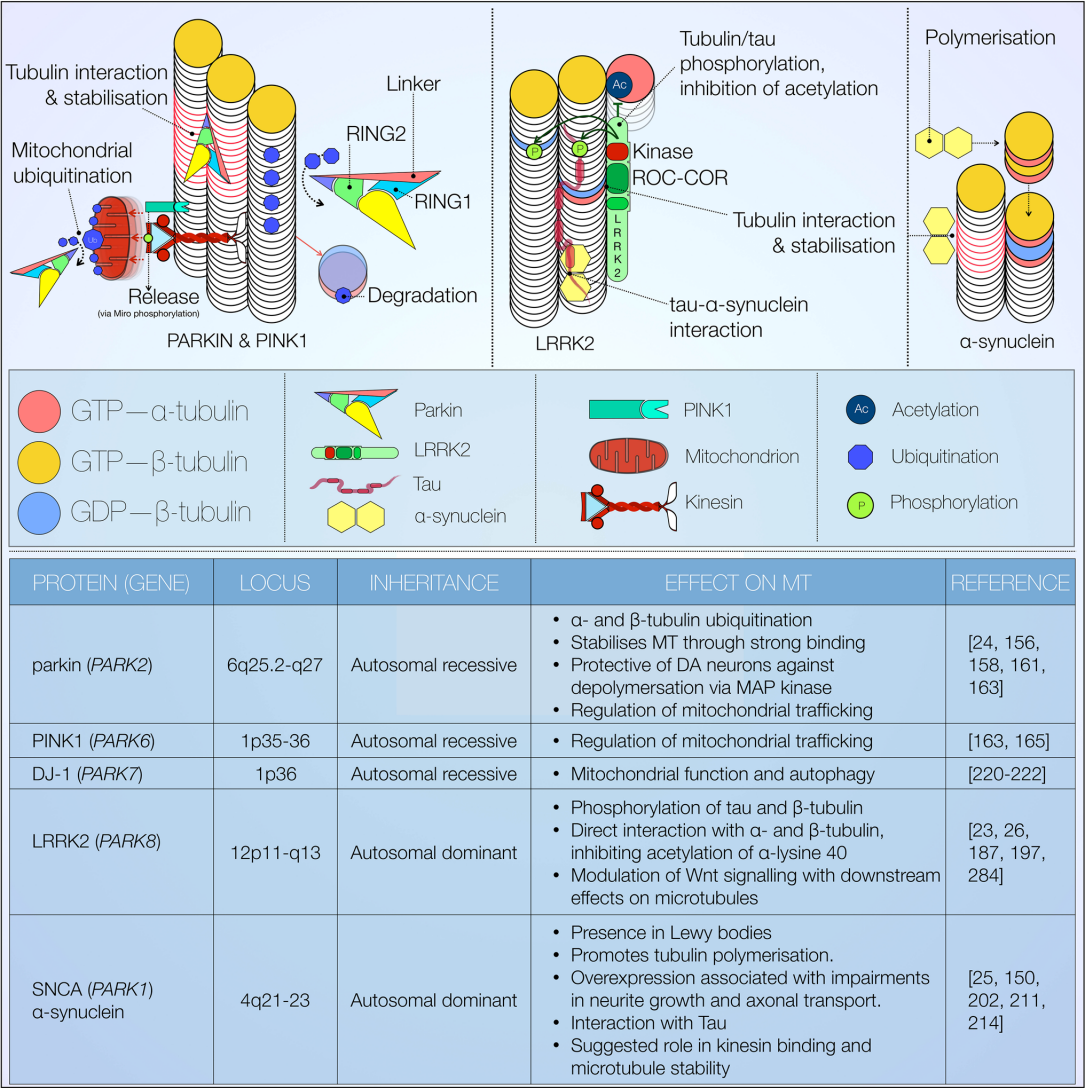
Effect of PD-linked proteins on microtubules. Overview of microtubule effects mediated by mutations in known PD-related genes, including inheritance patterns. All of the discussed proteins have demonstrated varying degrees of tubulin binding and the ability to modify microtubule function via several distinct mechanisms. Parkin structure adapted from [160]

A growing body of evidence suggests abnormal neuronal cytoskeleton assembly and function as key processes in neurodegenerative disease. Several proteins linked to neurodegenerative diseases including PD have been reported to bind tubulin directly and/or modulate microtubule stability (Fig. 5) [23–25, 156]. Here, we focus on evidence surrounding a number of key microtubule-interacting proteins—parkin, PTEN induced putative kinase 1 (PINK1), leucine-rich repeat kinase 2 (LRRK2) and α-synuclein (SNCA). Mutations in all corresponding genes cause autosomal forms of Parkinsonism [157]. Figure 5 summarises the major effects of PD-linked proteins on microtubules.

The *parkin* gene encodes a member of the E3 ubiquitin ligase family, which targets proteins for proteasomal degradation [158]. Exonic deletions in *parkin* were first identified in association to early onset autosomal recessive Parkinsonism in three unrelated Japanese families [159].

Physiological tubulin folding is dependent on the activity of multiple chaperone proteins. Dysfunctional protein folding machinery can induce toxic accumulation of misfolded tubulin heterodimers. Ren et al. reported that parkin enhances α/β-tubulin ubiquitination and clearance via a direct interaction [156]. In the same study, PD-associated *parkin* loss of function mutations was shown to abolish tubulin ubiquitination. This observation is suggestive of a potential pathogenic mechanism, although further investigation is required to verify these findings. In a later study, the authors reported high affinity parkin-tubulin binding via the linker (partially corresponding to RING0), RING1 and RING2 domains [24]. These interactions were subsequently shown to partially rescue colchicine-induced microtubule depolymerisation. Perhaps surprisingly, the microtubule-stabilising properties of parkin appear not to be compromised by PD-linked mutations [24]. A more recent publication further characterised the structure of human parkin, comprising four domains: UDP (Unique Parkin Domain, also known as RING0), RING1, IBR (In Between Ring Domain) and RING2 (Fig. 5) [160]. The authors reported that the UDP domain co-interacts with RING1 and RING2, and contains a putative phosphopeptide binding site. PD-causing mutations were subsequently mapped to this site [160]. Parkin was further shown to decrease MAP kinase activity, reducing microtubule depolymerisation [161]. This mechanism may be neuroprotective to midbrain dopaminergic neurons, a cell population highly vulnerable to microtubule depolymerisation [162]. Interestingly, PD-linked *parkin* mutations appear to abolish this effect [161].

Parkin has also been reported to regulate mitochondrial trafficking for subsequent degradation (mitophagy) [163, 164]. This mechanism is reportedly dependent on inhibitory phosphorylation of the mitochondrial Rho GTPase (Miro) [163, 165]. Miro, a calcium-binding GTPase, facilitates kinesin association with mitochondria, enabling their anterograde transport [166, 167]. Particularly, it is tethered to the mitochondrial surface via the adaptor protein milton. This interaction gives rise to a multi-protein complex binding kinesin heavy chain [168, 169]. Its inhibition results in release of mitochondria from kinesin (Fig. 5, middle left) [163, 166]. Miro phosphorylation is chiefly mediated by the serine/threonine kinase PINK1. Parkin-directed mitochondrial degradation thus appears to be PINK1-dependent [163, 170–172]. Wang et al. proposed an elegant functional hypothesis to account for this mechanism: PINK1/parkin-mediated mitochondrial transport arrest may serve as ‘quality control’, restricting motility to functional mitochondria [163]. It has been in fact previously shown that PINK1 is selectively cleaved into ∆N-PINK1 at active mitochondrial surfaces and subsequently degraded [173–175]. Interestingly, ∆N-PINK1 may also bind parkin to provide negative feedback inhibition of mitophagy. Together, these findings suggest mitochondrial activity may be an essential requirement for its efficient transport. This suggests that PD-linked loss of function mutations in *PINK1* and *parkin* allow aberrant transport of dysfunctional mitochondria, a process likely deleterious to neuronal viability in the long term. The precise molecular mechanism by which PINK1 and parkin regulate mitochondrial transport and ubiquitination endogenously remains elusive, however [176]. Upstream of PINK1, the serine/threonine MAP/microtubule affinity regulating kinase 2 (MARK2) has been identified as a regulatory enzyme [177]. Phosphorylation of the cleavage product ∆N-PINK1 by MARK2 was shown to promote anterograde mitochondrial motility [177, 178]. The authors also employ imaging to report PINK1 and MARK2 co-localisation with mitochondria, predominately within axons and dendrites. Whilst lacking a mitochondrial localisation signal, ∆N-PINK1 has also been shown to influence dendrite but not axonal morphology via interaction with MAP2 [179, 180].

Leucine-rich repeat kinase 2 (Fig. 5, centre) is another example of a microtubule-interacting protein with relevance to PD. Encoded by *LRRK2*, it is a large protein with overlapping functions in microtubule dynamic and intracellular signalling. LRRK2 contains kinase and GTPase (Roc-COR) domains, as well as multiple protein–protein interaction sites [181]. Mutations and variants in *LRRK2* are implicated in both autosomal dominant and sporadic PD [157, 182, 183]. All pathogenic mutations are located in the LRRK2 Roc-COR GTPase or LRRK2 kinase domain, and encompass single amino acid substitutions.

One of the most consistently observed effects of *LRRK2* mutations on the cytoskeleton is impaired neurite outgrowth [53, 184, 185]. Nonetheless, it is unclear if this is due to LRRK2 mediated changes in tubulin phosphorylation, tubulin acetylation, MAP phosphorylation and/or microtubule associated protein kinase regulation. Early in vitro experiments in HEK293 cells revealed LRRK2 co-localisation with β-tubulin [186]. We have also previously shown LRRK2 co-localisation with microtubules in growth cones [23, 187]. In this subcellular compartment, LRRK2 may also regulate the balance between stabilised and destabilised actin filaments [188]. Gillardon further reported that LRRK2 phosphorylates bovine brain β-tubulin [189]. Interestingly, phosphorylation was enhanced significantly by the pathogenic G2019S mutation in the LRRK2 kinase domain conferring increased LRRK2 autophosphorylation. We later demonstrated LRRK2 binding to the C-termini of only three out of eight β-tubulin isoforms possibly conferring some specificity in subcellular microtubule binding of LRRK2 [23]. Interestingly, this protein interaction takes place at the luminal microtubule surface in close proximity to the taxol binding site on β-tubulin and the lysine 40 α-tubulin acetylation sites. LRRK2 knockout was further shown to increase α-tubulin acetylation indicating that LRRK2 binding to microtubules might interfere with tubulin acetylation. In addition, we showed that the β-tubulin LRRK2 protein interaction strength was modulated by *LRRK2* mutations. Surprisingly, some mutations increased, whereas others decreased the protein interaction indicating that a fine regulation of this interaction might be required in order to avoid too much as well as too little α-tubulin acetylation [23]. Godena et al. more recently showed that both LRRK2 R1441C and Y1699C selectively bind deacetylated microtubules in vitro [26]. They suggested that this correlated to the observed mutation-dependent alterations in axonal transport in rat cortical neurons. Moreover, cells overexpressing mutant LRRK2 showed LRRK2 co-localisation with tubulin in filamentous structures. These mutants also altered axonal transport and induced motor impairments in *Drosophila* [26]. The LRRK2 kinase inhibitor IN-1 has now been reported to decrease α-tubulin acetylation and reduce microtubule stability in hybrid human cells [190]. A corresponding increase in free α-tubulin was also observed, consistent with microtubule depolymerisation.

In addition to tubulin, several lines of evidence suggest a functional interaction between LRRK2 and tau [191]. Tau neuropathology was reportedly observed in some human PD brains expressing the Y1699C, G2019S and I2020T *LRRK2* mutations [182, 192–194]. Age-related tau mislocalization and hyperphosphorylation have also been described in transgenic mice carrying the *LRRK2* G2019S or R1441G mutations [195, 196]. Importantly, Kawakami et al. described LRRK2-mediated tau phosphorylation at threonine 181 in vitro [184]. This reaction appears to be limited to tubulin-associated tau, and results in decreased tubulin binding. The authors propose a model, whereby LRRK2 controls tau release from microtubules through phosphorylation. The notion of a pathophysiological tau-LRRK2 interaction was corroborated in a further study by Bailey et al. [197]. They demonstrated LRRK2-mediated tau phosphorylation at a variety of residues in vitro. In addition, in a tau P301L mouse model, expression of *LRRK2* significantly increased deposition of insoluble, hyperphosphorylated tau [197]. LRRK2 has also been found to promote GSK3β activity and enhance tau phosphorylation [198, 199]. The above observations are in line with a tau mediated microtubule destabilising effect of LRRK2 mutations.

*SNCA*, encoding α-synuclein (Fig. 5, top right), was the first gene in which PD-linked dominant mutations were identified [200]. In addition, duplications and triplications of *SNCA* were also found to induce clinically diverse parkinsonian symptoms, suggesting a gene dose-dependent pathogenic effect [200, 201]. α-Synuclein accumulates and aggregates in Lewy bodies, the classical pathological hallmark of PD. These aggregates consist of a misfolded α-synuclein core surrounded by other proteins. A number of proteins have been found to co-localise with α-synuclein within Lewy bodies—namely tau, ubiquitin, tubulin as well as synaptic vesicle and stress-response proteins [202–204]. Some studies have also demonstrated presence of LRRK2, suggesting an interaction with α-synuclein [205, 206]. LRRK2 has further been suggested as upstream of either α-synuclein and/or tau phosphorylation [207]. This hypothesis is supported by LRRK2, α-synuclein and tau all representing genetic risk factors for sporadic PD [154, 205].

At present, neuronal α-synuclein function is understood incompletely. Several studies ascribe a physiological role of α-synuclein in the regulation of vesicle transport and dopamine release [208, 209]. α-Synuclein has been shown to interact with tubulin. Nonetheless, the effect of this interaction on tubulin polymerization and microtubule stability is less clear, with some studies suggesting a resulting increase in tubulin polymerisation whereas others suggest an impairment of polymerization [25, 210–212]. The result of α-synuclein treatment on neurite outgrowth is similarly inconclusive. Neurite outgrowth was reported to be enhanced in neurons treated with or overexpressing wild-type α-synuclein but not mutant forms of the protein [213]. By contrast, overexpression of wild-type and mutant α-synuclein has also been implicated in impaired neurite outgrowth, microtubule-dependent axonal transport, autophagy defects, and subsequent axonal degeneration [214]. Koch et al. suggest that the previously reported increase in neurite outgrowth [213] might in fact represent an acute response to exogenous α-synuclein expression. Conversely, chronically elevated levels of the protein would result in reduction of neurite length [214]. Interestingly, actin microfilaments and neurofilaments remained unaltered in another study reporting α-synuclein induced axonal degeneration [210]. To support this observation, the authors suggested a microtubule-selective mechanism for α-synuclein toxicity. On the other hand, tubulin oligomers reportedly stimulate α-synuclein fibrillogenesis [203].

Mutations in *SNCA* result in increased formation of α-synuclein β-sheet structures, resulting in the formation of toxic oligomers and mature fibrils [215]. In vivo design and biophysical analysis of fibril-promoting versus oligomer-promoting variants of α-synuclein reveals an important distinction. While α-synuclein oligomers correlate with neurotoxicity, the mature fibrils appear to be protective [216]. Accordingly, microtubule depolymerisation induced by mitochondrial dysfunction led to α-synuclein oligomerisation supporting the idea of an interdependence of changes in α-synuclein expression, microtubule depolymerisation and α-synuclein oligomerisation [217]. α-Synuclein has also been found to affect axonal transport, influencing microtubule stability and kinesin binding [211]. Consistent with their associated toxicity, α-synuclein oligomers significantly impair kinesin function. In a rat model of α-synucleinopathy, altered levels of presynaptic and axonal transport protein have also been observed [218].

Genome-wide association studies also highlighted a link between SNCA and MAPT, suggesting cooperation in idiopathic PD pathogenesis [154]. Synucleinopathies with tau-containing inclusions and vice versa have been reported, suggesting toxic interaction between tau and α-synuclein. Cross talk between these two proteins promotes synuclein fibrillization and increases tau phosphorylation in vitro and in vivo [219]. A direct interaction between α-synuclein and tau has also been shown [150]. Moreover, PD-inducing neurotoxins increase α-synuclein expression and downstream synuclein-dependent tau phosphorylation via PKA and GSK3β [152, 212]. The molecular interplay between α-synuclein, microtubules and tau remain a critical question.

Finally, the potential interplay between DJ-1 and microtubules is of note. DJ-1 function is still poorly characterised, and the majority of studies have focused on effects on mitochondrial function. Current evidence implicates loss of DJ-1 in mitochondrial fragmentation, autophagy and oxidative stress [220, 221]. PINK1/parkin-induced parkinsonian phenotypes are not rescued by *DJ*-*1* expression, suggesting potentially independent pathomechanisms [221]. A function for DJ-1 in microtubule dynamics has recently been revealed. siRNA-mediated knockdown of DJ-1 resulted in reduced β-tubulin III expression in neuroblastoma cells [222]. Importantly, a similar phenotype was observed in DJ-1 knockout mice. In both DJ-1 deficient model systems, microtubule polymerisation was reduced. Abnormal dendritic morphology was additionally observed in cultured striatal medium spiny neurons [222]. While these results offer promising insights into DJ-1 function on microtubules, extensive investigation into its potential relationship with previously discussed pathomechanisms is required.

The majority of experiments on the interaction of the cytoskeleton and familial PD proteins support the idea of a role of these proteins in the regulation of microtubule dynamics by different mechanisms including changes in PTMs of tubulin and tau, and changes in the activity of tau kinases. These observed changes can result in microtubule depolymerisation, subsequent impairment of axonal transport, synaptic dysfunction and eventually neurodegeneration. As the first insult seems to be a loss of fine regulation of the dynamic instability of microtubules the idea to use therapeutics strategies that restore this equilibrium is justified.

## Potential therapeutic strategies

Mounting evidence points to cytoskeletal dysfunction as an underlying mechanism in neurodegenerative diseases, including PD. Microtubule-oriented therapeutic approaches have been among the most successful in cancer therapy [223–225]. Several authors now also indicate microtubules as promising targets in neurodegenerative diseases [226, 227]. Here, we present potential therapeutic strategies, focusing on microtubule modulation and control of cellular signalling.

**Fig. 6:**
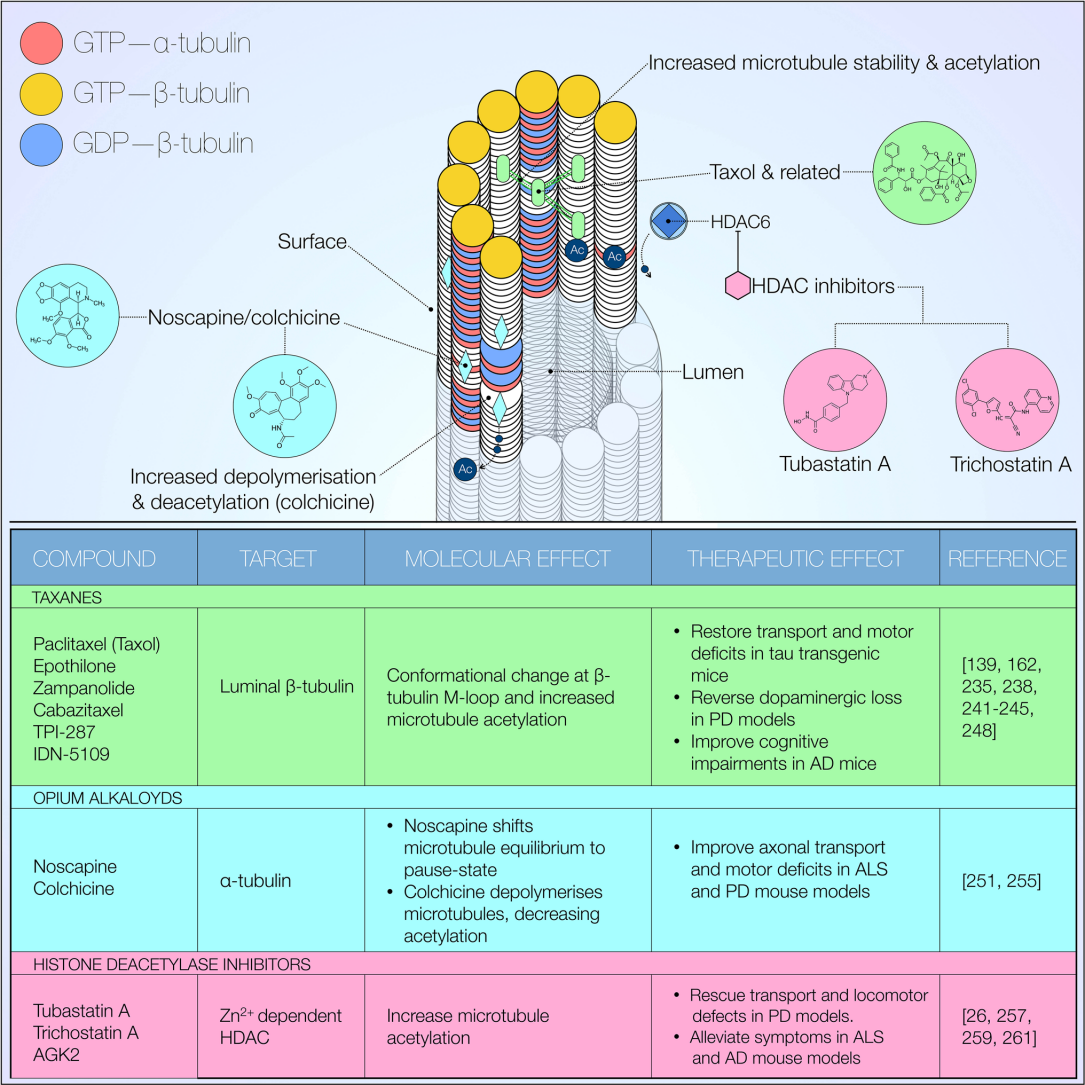
Microtubule-targeting agents (MTAs). Overview of microtubule targeting agents as potential therapeutic strategies. A variety of compounds has been shown to exert beneficial effects on microtubule PTMs, stability and transport. Additionally, the outlined agents are all reportedly able to produce improvements in PD-associated, model phenotypes. MTAs are also characterised by low toxicity at microtubule-modifying doses [Reference for chemical structures: https://www.ncbi.nlm.nih.gov/pccompound]

Microtubule-targeting agents (MTAs) are a group of compounds, which variably bind to and modify microtubule function (Fig. 6). Depending on their effect on polymerisation, they are classified as microtubule stabilising or destabilising agents. A number of MTAs are in various stages of preclinical and clinical development [225]. Examples discussed here include the taxanes, epothilone D and noscapine.

Members of the taxane family of compounds are amongst the better characterised MTAs. Paclitaxel (Taxol^®^) was first isolated from *T. brevifolia* in 1971 [228] and later identified as a microtubule binding, antimitotic agent [229–233]. Prolonged paclitaxel treatment has long been shown to induce microtubule stabilisation and α-tubulin acetylation [234]. This finding is suggestive of a mechanism whereby stabilised microtubules might facilitate acetyltransferase access to lysine 40. Given the importance of tubulin acetylation in recruitment of motor proteins [90, 101, 102], enhanced microtubule stability may constitute a requirement for efficient axonal transport. A definitive mechanism for paclitaxel-induced microtubule stabilisation was proposed in more recent years, ascribing its effect to luminal β-tubulin binding. Such interaction induces lateral protofilament stabilisation following a conformational change at the β-tubulin M-loop [235, 236]. The subsequently named taxane binding site has been the focus of extensive investigation into a number of compounds with similar binding properties. Two prominent examples are the epothilones (see below) and zampanolide, with X-ray crystallography confirming reversible and covalent binding to the taxane site, respectively [229, 237]. With relevance to neurodegeneration, selective dopaminergic loss in rotenone-induced Parkinsonism is reversed by paclitaxel [162, 235]. The outcomes of paclitaxel treatment on axonal transport are diverse, however, and still under debate. A study by Karbowski et al. has in fact shown that paclitaxel-induced microtubule stabilisation does not affect intracellular trafficking and distribution of mitochondria [238]. Conversely, paclitaxel restored alterations in fast axonal transport, microtubule detyrosination as well as motor deficits in tau transgenic mice [139]. An intriguing explanation for this effect was proposed by the authors, whereby paclitaxel does not reduce the pathological burden of tau inclusions per se. Rather, this compound acts as a functional replacement for sequestered tau [139]. Interestingly, the taxane binding site is also targeted by the microtubule-binding domain of tau, with paclitaxel inducing tau displacement from microtubules [236, 239]. Despite such effectiveness, a major challenge to paclitaxel administration is posed by its very poor penetrance of the blood–brain barrier [240]. To address this issue, synthetic paclitaxel derivates with an improved delivery profile have been developed. Examples include cabazitaxel, TPI-287, and IDN-5109 [241–245]. The paclitaxel-peptide conjugate, GRN1005, a paclitaxel-peptide conjugate, was also recently developed to enhance neuronal delivery. Following administration in patients with advanced solid tumours, the active paclitaxel-GRN1005 complex was detected within brain metastases [246].

The epothilone family of taxol-like compounds comprises blood–brain barrier-penetrant microtubule stabilising agents which have recently been a research focus in cancer and neurodegeneration [103, 240, 247]. Similarly to paclitaxel, their efficacy is thought to originate from functional replacement of tangle-sequestered tau. Long-term epothilone D (Epo D) treatment at subcytotoxic doses restored axonal morphology and microtubule density to wild-type levels in young PS19 tau transgenic mice. AD-like cognitive impairments associated with this murine model were also ameliorated [248]. Similar results were later obtained in aged PS19 mice, in which Epo D rescued axonal transport defects and improved cognition [249]. Both studies demonstrate the absence of dose-limiting side effects, indicating Epo D as a promising candidate for neurodegeneration therapy. Cartelli et al. later investigated a similar treatment regimen in a 1-methyl-4-phenyl-1,2,3,6-tetrahydropyridine (MPTP) murine model of PD [103]. These mice display nigrostriatal degeneration with early PTM alterations in α-tubulin. Dopaminergic loss, as well as the altered PTMs was both ameliorated following systemic, long-term Epo D injections [103]. The authors argue that disruption of neuronal microtubule dynamics may constitute an early, preclinical event in PD pathogenesis, supporting the notion of microtubule stabilisation as a valid therapeutic approach. A recent study has further highlighted the neuroprotective properties of Epo D [250]. In vitro modelling of axonal transection injury now demonstrates a dose-dependent enhancement of post-injury recovery. Particularly, Epo D improves axonal sprout number significantly while growth kinetics remains unaffected. The authors also report cultured neurons to remain viable throughout a wide dose range. This observation confirms the relatively low toxicity of Epo D doses at which microtubule-stabilising effects can be achieved.

Noscapine, an opium alkaloid with established antitussive effects [251], is another interesting candidate due to reported, albeit weak, microtubule-modifying properties [252–254]. While noscapine does not appear to significantly affect microtubule polymerisation or overall amounts of intracellular tubulin, it has been characterised as tubulin-binding compound in vitro [253, 255]. Additionally, a number of noscapine-mediated neuroprotective effects have been observed. In a SOD1 transgenic mouse model of ALS, microtubule and axonal transport deficits are reportedly improved following noscapine treatment [247]. Particularly, a significant reduction in microtubule turnover rate was observed in association with delayed disease onset and improved motor behaviour. Further experiments by this group later characterised axonal transport-mediated cerebrospinal fluid (CSF) secretion of a number of known cargo proteins, including α-synuclein and APP [256]. PD patients displayed CSF secretion profiles consistent with significant alterations in cargo transport dynamics. This observation was paralleled in MPTP-treated as well as A53T SNCA mutant mice. Interestingly, noscapine treatment significantly improved the CSF phenotype [256].

Considering the importance of tubulin acetylation in maintenance of intracellular transport [90, 101], pharmacological modulation of this PTM may prove useful in PD treatment. Histone deacetylase inhibitors (Fig. 6) have long been employed in psychiatry, and in more recent years as antimitotic agents. Because of the functional overlap of histone acetyltransferases in structural control of both chromatin and tubulin, the aforementioned class of compounds has indeed proven capable of altering microtubule function. Treatment of hippocampal neurons with trichostatin A, a histone deacetylase I-II inhibitor, has been shown to direct kinesin-1-mediated JIP1 transport to neurite tips [102]. A recent study characterised microtubule dysfunction in Rett syndrome, a rare neurodevelopmental condition [257]. In patient-derived fibroblasts, reduced α-tubulin acetylation was observed in correlation with increased expression of HDAC6. Additionally, microtubule polymerisation was markedly decreased. A similar phenotype was also observed in Rett syndrome mouse models. Administration of tubastatin A, an HDAC6 inhibitor, was then investigated. Within both model systems, pharmacological inhibition of HDAC6 restored α-tubulin acetylation levels. The polymerisation phenotype was also ameliorated, albeit to a more limited extent [257]. Taken together, these compelling data suggest targeting of deacetylated α-tubulin as a promising therapeutic strategy in Rett syndrome. Translating this approach to PD, however, demands further insight into a potential microtubule-mediated pathomechanism.

In this respect, the study by Godena et al. introduced in the previous section is of relevance [26]. *Drosophila* axonal transport and locomotor deficits induced by PD-associated *LRRK2* mutations were reversed by trichostatin A, tubastatin A or AGK2, a SIRT2 inhibitor [26]. As mentioned previously, abnormal filamentous LRRK2 was detected in marked co-localisation with tubulin. These aberrant structures were suppressed by deacetylase inhibitor treatment in HEK293 cells and rat cortical neurons. Furthermore, αTAT1 expression also prevents the mutant phenotype [26]. In accordance with these data, another study reports on a partial phenotype rescue in a 1-methyl-4-phenylpyridinium (MPP^+^)-induced zebrafish PD model [258]. This fish suffers from impaired locomotion and sensorimotor reflexes, as well as lowered cellular metabolic rate. Both tubastatin A and MS-275—a synthetic HDAC1 inhibitor—rescued the metabolic phenotype. Both inhibitors, however, failed to improve locomotion [258]. The authors thus highlight the need for further studies in models of genetically induced PD to clarify potential confounding factors. In the wider context of neurodegeneration, mouse models of ALS [259], AD [260] and Charcot–Marie–Tooth (CMT) disease [261] may also benefit from HDAC6 inhibition. In all cases, deacetylase inhibitor therapy resulted in symptomatic alleviation. However, it should be noted that oxidative stress induced by the neurotoxin 6OHDA also reduces deacetylase activity via inhibition of SIRT2 [94]. This finding indicates that therapeutic effects of deacetylase inhibition may be context-dependent. An emerging trend suggests that control over cellular signalling pathways upstream of microtubule function might also constitute an effective therapeutic approach in neurodegenerative disease. Here, we present evidence on two key pathways: neurotrophic factor-dependent and Wingless/Int-1 (Wnt) signalling.

Accumulating evidence increasingly supports modulation of neurotrophic factors (NTFs) as a potential strategy in protecting dopaminergic neurons from insult [262–264]. Jiang et al. evaluated the neuroprotective effects of three common NTFs: nerve growth factor (NGF), BDNF and glia cell line-derived neurotrophic factor (GDNF) [265]. The authors employed rotenone and colchicine-mediated modelling of dopaminergic loss in cultured midbrain neurons. These model systems display extensive microtubule depolymerisation and death of tyrosine hydroxylase-positive neurons. Such phenotype further reiterates the importance of a functional microtubule network in neuronal viability. In all cases, neurotrophic factor treatment significantly attenuated toxicity and restored microtubule polymerisation. The latter effect is ascribed by the authors to activation of microtubule-associated protein kinase kinase (MEK). Conversely, MEK inhibition abolished NTF-mediated neuroprotection [265].

To date, several clinical trials have investigated GDNF treatment in PD patients [262, 266]. However, results have so far been inconclusive [267]. In 2006, Salvatore et al. highlighted difficulties in midbrain drug delivery as a potential explanation for inefficacy of GDNF [268]. More recent work investigating viral vectors for sustained NTF delivery has offered promising results [269]. A number of studies have also investigated the effect of HDAC inhibitors on NTF gene expression. Valproate, known as antiepileptic agent, reportedly increases GDNF and BDNF expression in midbrain neuronal-astrocyte cultures by targeting HDAC2 [270]. Neuroprotection from MPP^+^ toxicity was achieved concomitantly. A similar outcome was later observed with an expanded range of HDAC inhibitors [271]. Due to the relatively broad spectrum of enzyme inhibition of HDAC inhibitors, it is worth pointing out that enhanced NTF gene expression might result from a histone-dependent mechanism unrelated to microtubules. A comparative analysis of expression profiles under a range of MTA treatments could offer insight into this mechanism.

Three independent highly conserved Wnt signalling pathways have been described: the canonical pathway plays key roles in gene expression via transcriptional activation of β-catenin, the noncanonical/planar cell polarity pathway (PCP) controls cytoskeletal rearrangement in development through activation of Rho GTPase and Jun N-terminal kinase (JNK) and the Wnt/Ca^2+^ pathway modulates cytosolic calcium levels [181, 272]. Although functionally distinguishable, all three share sensitivity to the Wnt family of ligands.

Wnt signalling exerts control over microtubule dynamics [181, 273] and midbrain dopaminergic neuronal development [274]. The pathway is generally well characterised in the context of nervous system development. Its finely regulated activity provides axonal guidance and remodelling, control of dendrite morphology and synapse formation [275, 276]. Interestingly, all of these mechanisms are heavily dependent on cytoskeletal function [277]. In this respect, Wnt-mediated regulation of cell polarity has been characterised in mouse embryonic fibroblasts [278]. The authors indicate that GSK3β, dishevelled (DVL) and axin—three key Wnt components—are required for translocation of the centrosome to the leading edge of polarising cells. Additionally, adenomatous polyposis coli (APC), a further Wnt protein, were previously revealed to accumulate and bind to microtubule plus ends [279]. Moreover, APC interaction with membrane components leads to microtubule polarisation [280]. In embryonic neuronal tissue, Wnt signalling appears to interact with actin-microtubule tethering mechanisms [281]. This process is reportedly mediated by microtubule actin cross-linking factor 1 (MACF1). Homozygous silencing of the corresponding gene is lethal early in embryonic development. The authors describe a phenotype similar to ablation of Wnt ligand production. Moreover, MACF1 was revealed to participate in translocation of key Wnt components to the membrane in a putative microtubule-dependent process [281].

Some of the PD-related proteins, discussed previously, have been reported to affect Wnt signalling—namely parkin [282], PINK1 [283] and LRRK2 [187, 284]. For the latter, multiple interactions have been described. LRRK2 appears to bind low density lipoprotein receptor-related protein 6 (LRP6), axin1, GSK3β, β-catenin and DVL 1-3 [187, 284]. Consequently, it was proposed to serve as a scaffolding protein facilitating Wnt signalling activation [284]. Indeed, expression of LRRK2 directly enhances β-catenin transcriptional activity [54, 284]. The aforementioned PD-associated, Roc-COR *LRRK2* mutations appear to partly abolish this effect while modulating LRRK2-LRP6 interaction [54]. Interestingly, Godena et al. reported on axonal transport disruption and locomotor defects induced by these mutations, as previously discussed [26]. By inference, microtubule/LRRK2/Wnt interplay might indeed constitute an additional candidate mechanism for molecular regulation of axonal transport [23, 191, 285]. Further investigation is required to establish the extent of this interaction, for instance, by determining whether the *LRRK2* mutation-mediated effects on Wnt signalling can be rescued by microtubule-stabilising therapy. Investigators also propose pharmacological modulation of the Wnt signalling pathways as a potential strategy in PD treatment [274, 286]. These pathways are extremely complex, however, and target diverse cellular mechanisms. Their functional heterogeneity is a potential confounding factor, and needs to be taken into account when investigating cell-wide effects of Wnt signalling modulation.

## Discussion and concluding remarks

We have discussed key evidence surrounding microtubule dysfunction in neurodegenerative disease, with particular focus on PD. A multitude of proteins and pathways of varying functional relationships with microtubules seem to be implicated in parkinsonian phenotypes. PD-linked proteins involved are associated with mitochondrial transport [163, 165], microtubule assembly and stability [25, 211], dynamics [187] and association of motor proteins [141]. Furthermore, their aberrant activity results in impairment of axonal transport, neurite outgrowth and generally induces morphological abnormalities in neurons [26, 54]. All of these proteins are able to interact with microtubules, either directly or via adaptor and motor proteins. Arguably, any protein which is transported along microtubules will ultimately bind them through a variety of mechanisms. In relation to PD, this line of reasoning warrants the following question: Is the specific function of the involved proteins the primary cause of toxicity, or do transport defects induce pathology downstream?

To initially address this issue, we propose a ‘traffic wave’ model of microtubule dysfunction in PD, and by extension, neurodegenerative disease. This constitutes an attempt to account for the diversity of microtubule-associated processes which lead to impaired neuronal function. Traffic wave disturbances in travel propagate and intensify retrogradely in relation to traffic direction. Generally, they result in perturbations significantly larger than the initial ‘insult’, and travel backwards for extended distances. We propose that a similar phenomenon, or rather the long-term accumulation of transportation defects, may ultimately result in (a) disruption of cytoskeletal architecture and (b) loss of functional localisation of any protein or organelle whose transport is secondarily delayed/affected.

There are a number of observations which lend credibility to this line of reasoning. As previously discussed, both tau and α-synuclein associate with microtubules and tau has been shown to promote axonal transport [25, 139, 140, 213]. Tau exerts an antagonistic action on motor protein binding. Its functional loss due to aggregation could therefore induce excessive motor protein binding. This may also subsequently result in higher amounts of motor-bound cargo. Accumulation of motor proteins may reduce transport speed and propagate retrogradely as a concentration gradient. Indeed motor protein gradients are known to directly determine the directionality of microtubule transport and movement [287]. The alternation between anterograde and retrograde transport is dependent on relative concentrations of dynein and kinesin. Uncoordinated directional switches caused by altered gradients may decrease transport efficiency. This might ultimately lead to an unpredictable array of functional consequences. One prominent example could be delay in delivery of functional mitochondria, depriving parts of the cell of efficient energy production. Considering that molecular motors are dependent on local ATP gradients [115], retrogradely propagating changes in energy availability may also progressively reduce the ability of microtubules to deliver cargo to subcellular compartments. In addition, changes in tau or α-synuclein phosphorylation state are known to affect microtubule polymerisation [137]. A slower polymerising microtubule may induce accumulation of anterograde cargo at the plus end. This might in turn propagate backwards along the microtubule and further impair transport.

The discussed LRRK2-mediated effects might also aid corroboration of this model. For example, Godena et al. demonstrated a mutation-dependent decrease in axonal trafficking [26]. At the same time, however, mutant LRRK2 was seen in close association to microtubules, far more so than WT LRRK2. It may be argued that axonal transport is slowed down by actual physical interference of mutant LRRK2 with the microtubule network which, in turn, retrogradely propagates delays in transport. Given the discussed importance of fine regulation of tubulin PTMs, a cargo-crowded microtubule might indeed be more impervious to enzymatic access. Changes in acetylation or phosphorylation status, for instance, might propagate retrogradely and ultimately destabilise microtubules.

The current outlook on therapeutic strategies is also of relevance. Most, if not all microtubule-oriented approaches share a common principle: stabilisation of microtubules appears to be beneficial [226, 227]. While these agents do not necessarily target the pathological burden of PD-linked proteins—e.g., aggregate formation—they do restore appropriate axonal transport and consequently ensure cargo delivery. Their beneficial effects might therefore be due to maintenance of functional localisation of virtually any protein transported along microtubules.

In conclusion, intracellular transport alterations may induce secondary damage not always in functional relation to the protein originally causing the defects. Amongst all cell types, neurons are some of the most heavily dependent on a functional microtubule network. PD-linked proteins may, however diverse, be dependent on a shared, microtubule-dependent pathway. Therefore, development of effective therapies aimed at maintaining a functional cytoskeleton may constitute an effective approach.
